# Extracellular volume-guided late gadolinium enhancement analysis for non-ischemic cardiomyopathy: The Women’s Interagency HIV Study

**DOI:** 10.1186/s12880-021-00649-6

**Published:** 2021-07-27

**Authors:** Yoko Kato, Jorge R. Kizer, Mohammad R. Ostovaneh, Jason Lazar, Qi Peng, Rob J. van der Geest, Joao A. C. Lima, Bharath Ambale-Venkatesh

**Affiliations:** 1 Department of Cardiology, Johns Hopkins University, Baltimore, MD USA; 2 Cardiology Section, San Francisco Veterans Affairs Health Care System, and Departments of Medicine, Epidemiology and Biostatistics, University of California San Francisco, San Francisco, CA USA; 3 SUNY Downstate Medical Center, New York, NY USA; 4 Albert Einstein College of Medicine, New York, NY USA; 5 Department of Radiology, Leiden University Medical Centre, Leiden, The Netherlands; 6 Division of Radiology, Johns Hopkins University School of Medicine, 600 N Wolfe Street MR 110, Baltimore, MD 21287 USA

**Keywords:** ECV-guided LGE analysis, Magnetic resonance imaging (MRI), Late gadolinium enhancement (LGE), Extracellular volume fraction (ECV), Non-ischemic LGE, Human immunodeficiency virus (HIV), Scar quantification

## Abstract

**Background:**

Quantification of non-ischemic myocardial scar remains a challenge due to the patchy diffuse nature of fibrosis. Extracellular volume (ECV) to guide late gadolinium enhancement (LGE) analysis may achieve a robust scar assessment.

**Methods:**

Three cohorts of 80 non-ischemic-training, 20 non-ischemic-validation, and 10 ischemic-validation were prospectively enrolled and underwent 3.0 Tesla cardiac MRI. An ECV cutoff to differentiate LGE scar from non-scar was identified in the training cohort from the receiver-operating characteristic curve analysis, by comparing the ECV value against the visually-determined presence/absence of the LGE scar at the highest signal intensity (SI) area of the mid-left ventricle (LV) LGE. Based on the ECV cutoff, an LGE semi-automatic threshold of n-times of standard-deviation (n-SD) above the remote-myocardium SI was optimized in the individual cases ensuring correspondence between LGE and ECV images. The inter-method agreement of scar amount in comparison with manual (for non-ischemic) or full-width half-maximum (FWHM, for ischemic) was assessed. Intra- and inter-observer reproducibility were investigated in a randomly chosen subset of 40 non-ischemic and 10 ischemic cases.

**Results:**

The non-ischemic groups were all female with the HIV positive rate of 73.8% (training) and 80% (validation). The ischemic group was all male with reduced LV function. An ECV cutoff of 31.5% achieved optimum performance (sensitivity: 90%, specificity: 86.7% in training; sensitivity: 100%, specificity: 81.8% in validation dataset). The identified n-SD threshold varied widely (range 3 SD–18 SD), and was independent of scar amount (β = −0.01, *p* = 0.92). In the non-ischemic cohorts, results suggested that the manual LGE assessment overestimated scar (%) in comparison to ECV-guided analysis [training: 4.5 (3.2–6.4) vs. 0.92 (0.1–2.1); validation: 2.5 (1.2–3.7) vs. 0.2 (0–1.6); *P* < 0.01 for both]. Intra- and inter-observer analyses of global scar (%) showed higher reproducibility in ECV-guided than manual analysis with CCC = 0.94 and 0.78 versus CCC = 0.86 and 0.73, respectively (*P* < 0.01 for all). In ischemic validation, the ECV-guided LGE analysis showed a comparable scar amount and reproducibility with the FWHM.

**Conclusions:**

ECV-guided LGE analysis is a robust scar quantification method for a non-ischemic cohort.

*Trial registration* ClinicalTrials.gov; NCT00000797, retrospectively-registered 2 November 1999; NCT02501811, registered 15 July 2015.

**Supplementary Information:**

The online version contains supplementary material available at 10.1186/s12880-021-00649-6.

## Background

Replacement fibrosis of left ventricular (LV) scar quantified by the amount of late gadolinium enhancement (LGE) has been shown to be a better predictor of the risk of incident adverse clinical events than the presence/absence of LGE alone in different cardiac diseases [1–4]. However, quantification of LGE scar is challenging when the scar distributions are diffuse and patchy, which is typical in non-ischemic cardiomyopathy such as myocarditis [5–7], cardiomyopathies [8–11], and in human immunodeficiency virus (HIV) cohorts [12–16]. As a result, the quantification of scar mass or even the identification of LGE can present a huge challenge in non-ischemic cases.

The most recent SCMR task force recognized that there is not enough evidence to provide a cut-off for non-ischemic LGE scar quantification [17]. A number of LGE scar quantification methods with semi-automated thresholds have been proposed and utilized [5, 9, 17–23]. These methods use a fixed calculation formula to define a signal intensity (SI) threshold value above which the tissue is identified as a myocardial scar. Their performance is well recognized in ischemic cases while in non-ischemic cases, it is not infrequent that substantial manual correction is needed after semi-automated thresholding. Therefore, by default, manual scar delineation remains the current standard of reference in non-ischemic cases [17]. However, manual analysis is challenging as the non-ischemic patchy fibrosis presents a rather low contrast on LGE when compared to the ischemic dense fibrotic scar [24].

T1 mapping may be more accurate in the assessment of diffuse and patchy myocardial fibrosis [25–32]. Extracellular volume fraction (ECV) is robust across different field strengths and acquisitions [13, 15, 33, 34]. On the other hand, current T1 mapping still has notable drawbacks, including the time required for whole LV coverage as compared to 3D LGE techniques and lower spatial resolution than LGE. Given the advantages and limitations of T1 mapping and LGE, we hypothesized that combining these two images in a complementary manner may achieve a more robust and comprehensive LV scar assessment, particularly applicable to the non-ischemic cases. We, therefore, set out to establish ECV criteria to differentiate LGE scar from non-scar that is utilized for the ECV-guided LGE analysis and compare the performance of this approach with the conventional manual LGE analysis in a non-ischemic cohort of women with or at risk for HIV infection. Additionally, the developed ECV-guided LGE analysis technique was validated in a different subset of 20 non-ischemic and 10 ischemic cases.

## Materials and methods

### Cohort

**Fig. 1:**
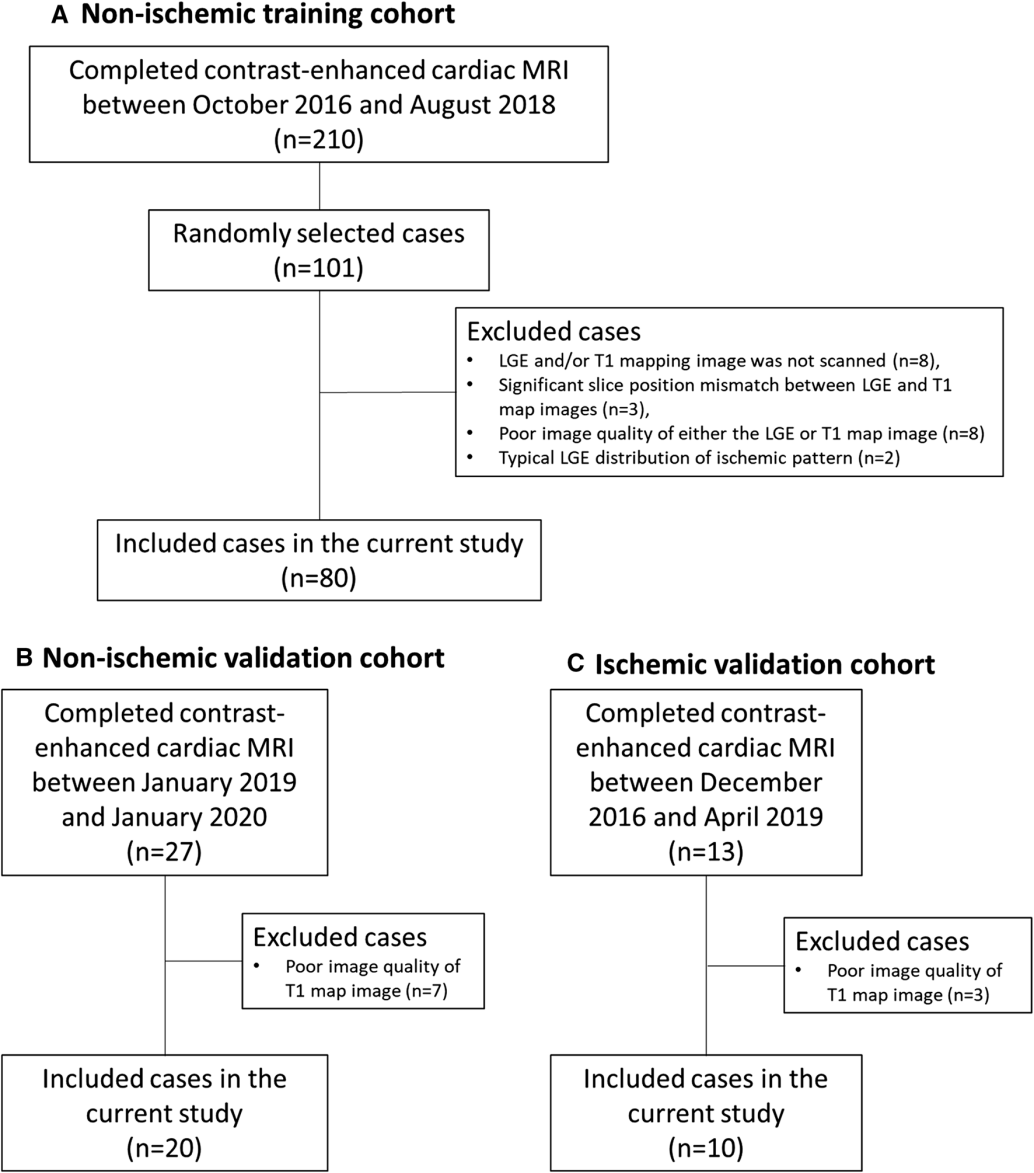
Study flowcharts. **A** Non-ischemic training cohort. **B** Non-ischemic validation cohort. **C** Ischemic validation cohort. **A** Of the 210 participants that completed contrast-enhanced cardiac MRI between October 2016 and August 2018, a sub-sample of 101 women was randomly selected for the present study. Twenty-one of them were excluded from image analysis due to the reasons listed in the flowchart. Overall 80 participants were included in the non-ischemic training cohort. **B** Of the continuous 27 contrast-enhanced cardiac MRI between January 2019 and January 2020, seven of them were excluded from the image analysis due to the image quality issue. Overall 20 cases were included in the non-ischemic validation cohort. **C** Of the continuous 13 contrast-enhanced cardiac MRI between December 2016 and April 2020, three of them were excluded from the image analysis due to the image quality issue. Overall 10 cases were included in the ischemic validation cohort. *MRI* magnetic resonance imaging

The study flowcharts are presented in Fig. 1. This study consisted of three cohorts of non-ischemic training cohort, non-ischemic validation cohort, and ischemic validation cohort. The current MRI study was planned as an ancillary study of The Women’s Interagency HIV Study (WIHS), which is a multicenter study that enrolled women with, or at risk for, HIV infection in 1994–1995 and 2001–2002 at six U.S. sites. The study participants were enrolled in three stages at sites across the United States, with HIV-positive and HIV-negative recruited from the same clinics to ensure a similar sociodemographic and behavioral risk factor profile [35–37]. The non-ischemic training cohort participants in the presented study were recruited from two specific sites of WIHS study. Of 619 participants in active follow-up at both sites, 210 completed contrast-enhanced cardiac MRI between October 2016 and August 2018. Among these participants, a sub-sample of 101 women was randomly selected for the present study. Twenty-one of them were excluded from image analysis because of missing LGE and/or T1 mapping image data (n = 8), unacceptable slice position mismatch between LGE and T1 map images (n = 3), poor image quality of either the LGE or T1 map image (n = 8), or typical LGE distribution of ischemic pattern (n = 2). The non-ischemic validation cohort consisted of 20 cases from the same WIHS study, but the participants were enrolled and underwent cardiac MRI in a different site from the two sites of the training cohort. Their MRI scans were performed between January 2019 and January 2020. The ischemic validation cohort consisted of ten ischemic heart failure cases enrolled in an external cohort study entitled Combination of Mesenchymal and c-kit + Cardiac Stem Cells As Regenerative Therapy for Heart Failure (CONCERT-HF) [38], the details of which are summarized in Supplement 1 within the Additional file 1.

In both clinical trials from which the non-ischemic and the ischemic cohorts originated (non-ischemic [35–37]; ischemic [38]), all the cardiac MRI studies were approved by the institutional review boards (IRB) of each participating field center and all participants signed informed consent. Both studies complied with the World Medical Association's Declaration of Helsinki and registered with ClinicalTrials.gov (non-ischemic: number NCT00000797; ischemic: NCT02501811). The current study is planned and conducted under our IRB approval with the application number IRB00116189 for the non-ischemic cohort; IRB00089436 for the ischemic cohort.

### Cardiac MRI protocol

A standard Cardiac MRI protocol including LGE and T1 mapping was performed using two 3.0 Tesla magnets (Achieva and Ingenia, Philips Healthcare, Best, the Netherlands) at the Albert Einstein College of Medicine/Montefiore Medical Center (for the training cohort) and University of California Saint Francisco for the validation cohort. The LGE images were acquired 13–20 min after intravenous administration of 0.2 mmol/kg gadopentetate meglumine (Dotarem, Guerbet, Roissy, France) using a standard two-dimensional segmented phase-sensitive inversion recovery (PSIR) gradient echo sequence to cover the entire LV. The short axis T1 mapping images were acquired in one slice at the mid-LV level before and 20 min after contrast administration with 3(3)3(3)5 Modified Look-Locker Imaging (MOLLI) sequence [39] or the recently proposed 5s(3s)3s and 4s(1s)3s(1s)2s schemes [40]. All images were motion-corrected before analysis. The image acquisition parameters are documented in  Supplement 2 within the Additional file 1. ECV maps were calculated from pre- and post-contrast T1 values of the blood pool, myocardium, and hematocrit [28]. The MRI protocol of the ischemic cohort is summarized in Supplement 1 within the Additional file 1.

### Image analysis

Two experienced observers blinded to HIV status (Y. K. for T1 and LGE analysis, over 8 years of cardiac MRI experience; E. C. for T1 and LGE analysis, over 10 years of experience) performed image analysis using Medis Suite 2.1.12.6 for T1 mapping and QMass MR 7.5 for LGE (both Medis Medical Imaging Systems, Leiden, the Netherlands). The contours of the LGE and T1 images were prepared in advance to the scar quantification. On LGE images, epicardial and endocardial contours were manually traced for all the slices of the stack of short-axis LGE images. Then in the mid-LV slice corresponding to the ECV slice, remote myocardium and high SI area were automatically detected and assigned region of interests (ROIs) by the software, based on the minimum and maximum areas of signal intensity [41]. On T1 images, one set of epicardial and endocardial contours were manually traced sparing the edge of the myocardium to avoid the effect of misregistration errors. The ECV value of each pixel was automatically calculated from the inversion recovery curve on the workstation and the resulting ECV map was utilized.

An LGE scar quantification method of ECV-guided LGE analysis which applies ECV criteria and optimizes the n-SD threshold in the individual case was developed and compared with the conventional analysis methods of manual analysis (in the non-ischemic cohort) or with full-width half-maximum (FWHM) (in the ischemic cohort) [17–23]. These LGE scar quantifications were performed separately with at least 2 weeks between the reads. LGE analysis was performed on the PSIR images and was reported in global and segmental levels using the AHA 17 segment model [42].

### Preparation of the ECV criteria to detect LGE scar

ECV cutoff value to differentiate LGE scar/non-scar was investigated on the 80 training cohort subjects. First, the highest SI ROI on the mid-LV LGE was automatically detected by the software. The user visually inspected the area of highest SI ROI to determine if this corresponded to a scar or non-scar such as artifact, through-plane motion, partial volume effect, etc. The ECV value of the corresponding location (i.e., the highest SI ROI area on the LGE) was referenced from the ECV map. Then, a single ECV cutoff to differentiate LGE scar/non-scar was identified from receiver-operating characteristic (ROC) curve analysis. Furthermore, the same procedure to detect native T1 (nT1) cutoff was performed as a means to compare the performance of scar differentiation with that from ECV.

### ECV-guided LGE analysis

**Fig. 2:**
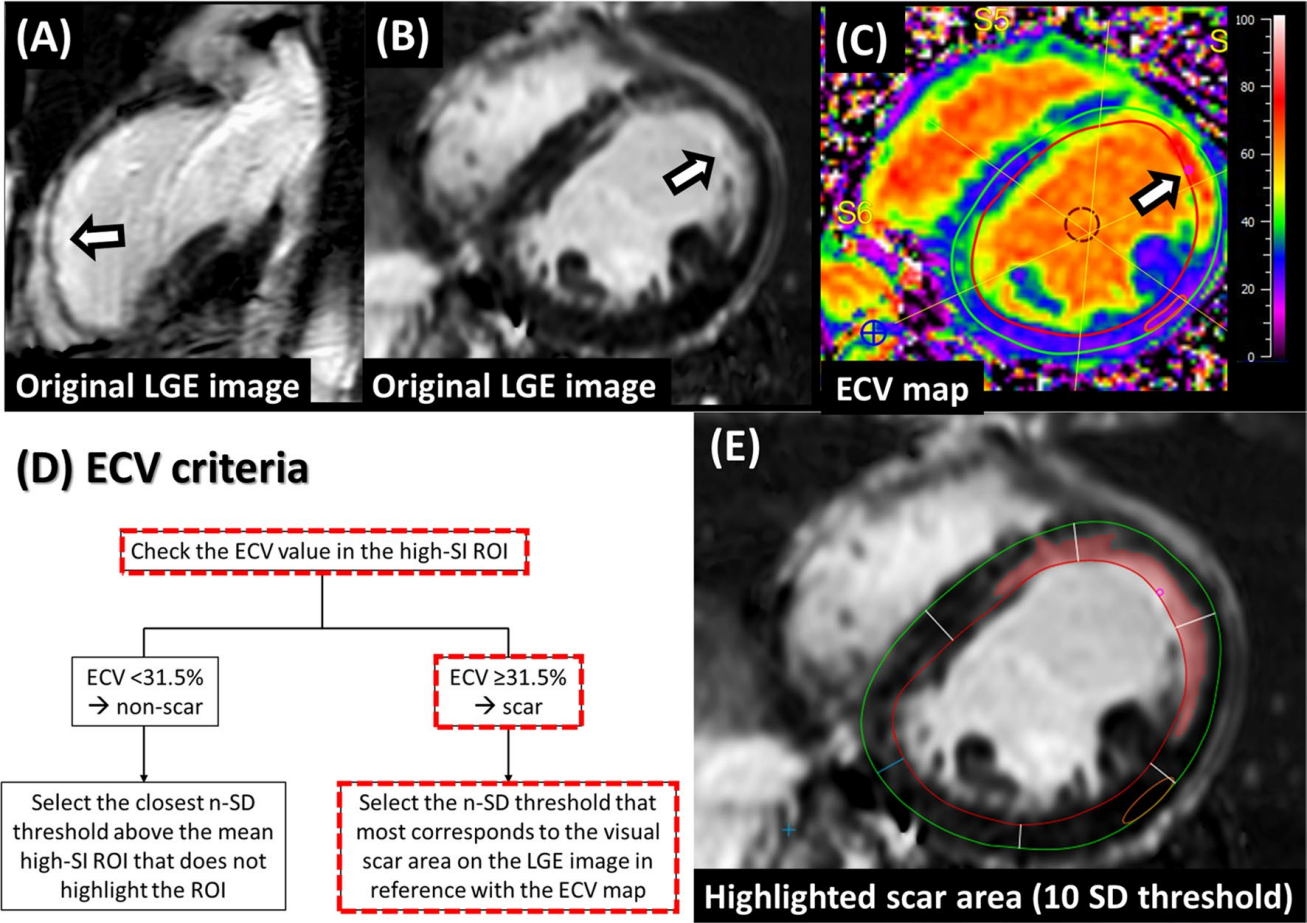
A representative case of ECV-guided LGE analysis. **A** Original LGE image at the 2-chamber view. **B** Original LGE image at the mid-LV slice. **C** ECV map. **D** ECV criteria flowchart. **E** LGE image at the mid-LV slice with highlighted scar area. A high SI was observed in the anterior wall (**A**, **B**, arrows). The ECV value at the corresponding location was 69.3%, which was higher than 31.5% (**C**, arrow). Based on the ECV criteria flowchart, the high SI area was judged as scar (**D**, red dotted line boxes). Then, the optimal n-SD threshold was selected on the LGE image in reference with the ECV map. In this case, the optimal threshold was 10SD (**E**). The selected threshold was propagated to other slices on the LGE image. *ECV* extracellular volume, *LGE* late gadolinium enhancement, *SI* signal intensity, *ROI* region of interest, *SD* standard deviation, *LV* left ventricle, *RV* right ventricle

**Fig. 3:**
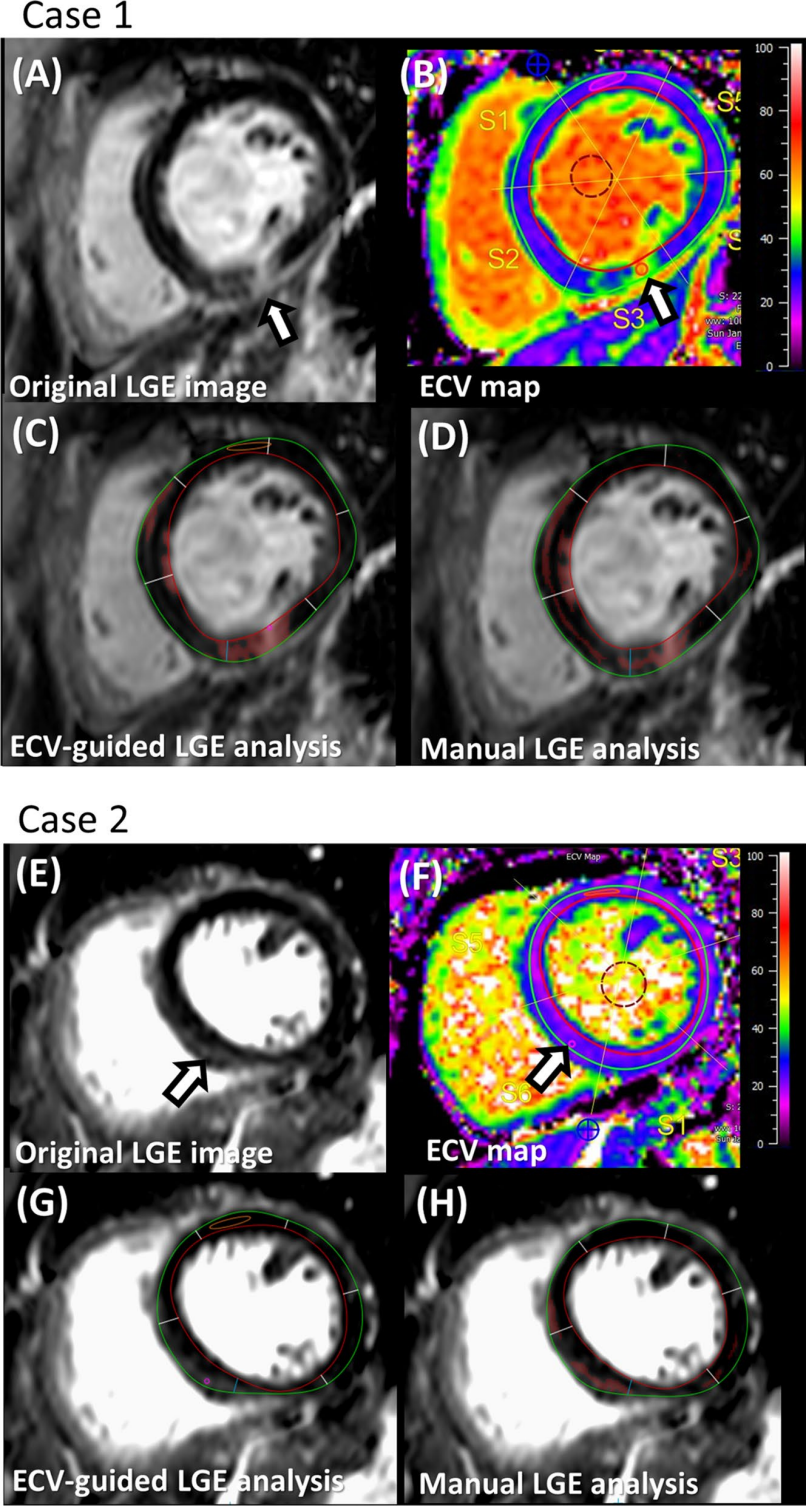
Representative cases presented with or without scar on ECV-guided LGE analysis. **A**–**D** Case 1, which presented with scar on ECV-guided LGE analysis. **A**, Original LGE image at the mid-LV slice. **B** ECV map, **C** LGE image at the mid-LV slice with highlighted area by the ECV-guided LGE analysis. **D** LGE image at the mid-LV slice with the manually highlighted area. **E**–**H** Case 2, which presented without scar on ECV-guided LGE analysis. **E** Original LGE image at the mid-LV slice. **F** ECV map. **G** LGE image at the mid-LV slice with no highlighted area by the ECV-guided LGE analysis. **H** LGE image at the mid-LV slice with the manually highlighted area. (Case 1) A high SI was observed in the inferior wall (**A**, arrow). The ECV value at the corresponding location was 58.3%, which was higher than 31.5% (**B**, arrow). Based on the ECV criteria , the high SI area was judged as a scar. The optimal threshold of 11SD was selected and highlighted the myocardium (**C**). The manual analysis also highlighted the corresponding area (**D**). (Case 2) A high SI was observed in the inferoseptum (**E**, arrow). The ECV value at the corresponding location was 26.5%, which was lower than 31.5% (**F**, arrow). Based on the ECV criteria , the high SI area was judged as a non- scar. The optimal threshold of 13SD was chosen which did not highlight the myocardium (**G**). Meanwhile, the manual analysis highlighted the myocardium (**H**). *ECV* extracellular volume, *LGE* late gadolinium enhancement, *LV* left ventricle, *SI* signal intensity, *ROI* region of interest, *SD* standard deviation

The scar presence/absence was first judged for the high-SI ROI by application of the ECV criteria and then the corresponding n-SD threshold was identified in reference to the ECV value of the corresponding location. These procedures were performed on the mid-LV slice which was the same slice level as the T1 map image. There were two scenarios: (1) If the high-SI ROI was judged as a scar (i.e., ECV value was above the ECV cutoff), then the optimal threshold (n-times of SD of remote myocardial SI, n-SD) above the remote myocardial mean SI was identified visually to delineate the scar area on the LGE slice. The ECV map was used for visual reference in addition to the LGE image to delineate a scar extent. (2) If the high-SI ROI was judged as a non-scar (i.e., ECV value was below the ECV cutoff), the closest threshold (n-SD) that does not highlight the high-SI ROI was selected. In both scenarios, the selected n-SD threshold was propagated to other slices on the LGE image. Acquisition-related artifacts, if present, were visually detected and erased. The scar mass (g) was recorded, and the final scar amount (%) was calculated from the scar mass (g) and LV mass (g). Representative cases are presented in Figs. 2 and 3.

### Statistical analysis

Data distribution was confirmed with histograms and Skewness/Kurtosis tests. Continuous data are expressed as mean ± SD or, and highly skewed as median (first and third quartiles). Wilcoxon rank-sum test and Wilcoxon signed-rank test were used as appropriate to compare the results between the two groups. Chi-square test was used to compare the LGE detection rate between two groups. Simple linear regression analysis was performed to investigate the association between the optimized threshold (n-SD) and the scar amount (%). The correlation between the two MRI indices was investigated with Spearman’s test. The ECV or nT1 cutoff value to differentiate LGE scar and non-scar was identified from the ROC curve analysis. The area under the curve (AUC) and the sensitivity, specificity, PPV, and NPV at the selected cut-off values are reported. For the inter-method scar amount agreement and the intra- and inter-observer reproducibility analysis, concordance correlation coefficients (CCC) (with CCC < 0.40 = poor; 0.40 ≤ CCC ≤ 0.75 = fair to good; 0.75 < CCC = excellent) and 95% limits of agreement (LoA) by the Bland–Altman analysis were investigated. Statistical significance was defined by a two-tailed *P* < 0.05. Power calculation was performed a priori to identify the number of cases to develop the ECV criteria to differentiate LGE scar from non-scar. With a type I error of 0.05 and Type II error of 0.20 to achieve an AUC of 0.7, with a null hypothesis of 0.5 and simulating the rate of positive LGE at the high SI ROI from 30 and 70%, a sample size range between N = 62 (with LGE positive rate of 50%) to N = 77 (with LGE positive rate of 30%) was identified. All analyses were conducted by Y.K. in STATA (Version 15.1, StataCorp, College Station, Texas, USA).

## Results

### Participant characteristics

The participant characteristics of the 80 non-ischemic training cohort, 20 non-ischemic validation cohort, and the 10 ischemic validation cohort are summarized in Table 1. In brief, the non-ischemic groups were all-female cohorts with HIV positive rates of 73.8% (training cohort) and 80% (validation cohort) while the ischemic group was an all-male cohort with significantly reduced LV ejection fraction (LVEF) .﻿ The LGE in the non-ischemic groups was diffuse and patchy with lower contrast (Fig. 3) when compared to the dense and focal ischemic scar (Fig. 2). The LGE in the non-ischemic group was mainly observed in the anteroseptum and inferoseptum in the basal to mid slices and in the basal inferior wall.

**Table 1:** Participant characteristics

Participant characteristics	Non-ischemic training cohort	Non-ischemic validation cohort	Ischemic validation cohort
N	80	20	10
Age (years old)	51.9 ± 8.7	56.1 ± 6.3	63.5 (59–75)
Female, n (%)	80 (100)	20 (100)	0 (0)
*Ethnicity distribution, n (%)*			
African American	51 (63.8)	11 (55.0)	N/A
Hispanic	25 (31.3)	1 (5.0)	N/A
Others	4 (5.0)	8 (40.0)	N/A
HIV positive participant, n (%)	59 (73.8)	16 (80.0)	N/A
LVEF (%)	56.0 ± 5.1	58.7 ± 5.6	37.1 (30.1–38.0)
LVEF < 50% case distribution, n (%)	10 (12.5)	1 (0.5)	10 (100)

Data are expressed as mean ± SD, or when highly skewed, as median (first and third quartiles), or in the exact number and the percentage

*MRI* magnetic resonance imaging, *HIV* human immunodeficiency virus, *LVEF* left ventricular ejection fraction, *N/A* not available

### ECV-guided LGE analysis in the non-ischemic training cohort

#### ECV criteria development

**Fig. 4:**
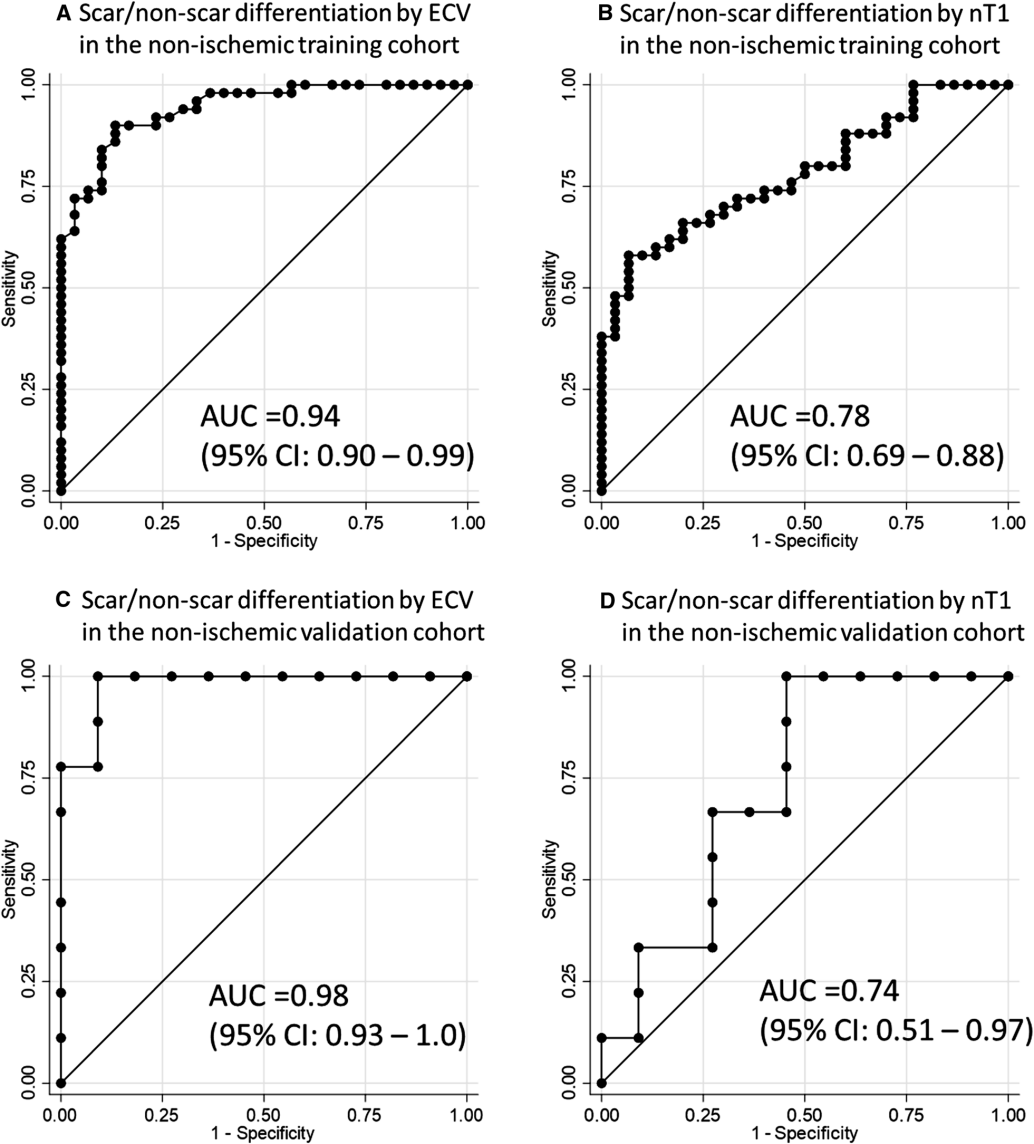
Comparison between the scar differentiation performance of ECV and nT1 in the non-ischemic training cohort and validation cohort. **A** Scar/non-scar differentiation performance of ECV in the non-ischemic training cohort. **B** Scar/non-scar differentiation performance of nT1 in the non-ischemic training cohort. **C** Scar/non-scar differentiation performance of ECV in the non-ischemic validation cohort. **D** Scar/non-scar differentiation performance of nT1 in the non-ischemic validation cohort. In both cohorts, ECV presented a better performance of scar/non-scar differentiation than nT1. In the training cohort, the derived ECV cutoff of 31.5% achieved sensitivity of 90%, specificity of 86.7%, PPV of 91.8%, and NPV of 83.9% (**A**) while the derived nT1cutoff of 1317 ms achieved sensitivity of 68%, specificity of 70%, PPV of 79.1%, and NPV of 56.8% (**B**). In the validation cohort, the ECV cutoff of 31.5% excellently differentiated scar/ non-scar (sensitivity 100%, specificity 81.8%, PPV 81.8%, and NPV 100%) (**C**) while the nT1 cutoff of 1317 ms presented a fair performance (sensitivity 33.3%, specificity 90.9%, PPV 75%, and NPV 62.5%) (**D**). *ECV* extracellular volume, *nT1* native T1, *AUC* area under the curve, *CI* confidence interval, *PPV* positive predictive value, *NPV* negative predictive value

The averaged ECV value corresponding to the high SI ROI was 34.0 ± 6.6 (%), which was higher than the global ECV value at the mid-LV slice of 27.1 ± 2.8 (%) (*P* < 0.01). In the visual assessment, 50 out of the 80 cases were judged as a scar. The ROC curve presented the AUC of 0.94 (95% CI 0.90–0.99). The single ECV cutoff identified from the ROC curve to differentiate scar from non-scar was 31.5%. Forty-nine cases (61.2%) fit into the ECV ≥ 31.5% scar category while 31 cases (38.8%) fit into the non-scar category of ECV < 31.5%. Overall, the ECV cutoff value of 31.5% achieved a sensitivity of 90%, specificity of 86.7%, PPV of 91.8%, and NPV of 83.9%. In the nT1 investigation, the averaged nT1 value corresponding to the high SI ROI was 1339 ± 94 (ms). The ROC curve of nT1 against visual scar/non-scar presented the AUC of 0.78 (95% CI 0.69–0.88). The nT1cutoff of 1317 (ms) achieved a sensitivity of 68%, specificity of 70%, PPV of 79.1%, and NPV of 56.8%. Based on these results, the ECV cutoff was selected to guide the LGE analysis (Fig. 4A, B).

#### The optimization of the n-SD threshold in the non-ischemic training cohort

The optimal threshold (n-SD) on average was 7.3 ± 2.9 (SD), which showed a wide range from 3SD minimum to 18SD maximum. The threshold value (n-SD) was not associated with the global LGE scar amount (%) assessed in ECV-guided LGE analysis (β = −0.01, *P* = 0.92) and furthermore, there was no difference between LGE positive (n = 61) and negative (n = 19) cases. (LGE positive vs. negative = 7.1 ± 2.8 SD vs. 7.7 ± 3.4 SD, *P* = 0.51). Among the 31 cases in which high-SI ROI was judged as non-scar and therefore no scar in the mid-LV slice, 11 of them presented scar in other slices after the propagation of the selected threshold. These 11 cases showed typically a small global scar percentage of 0.46 (0.15–1.1) %.

#### Inter-method agreement in the non-ischemic training cohort

**Fig. 5:**
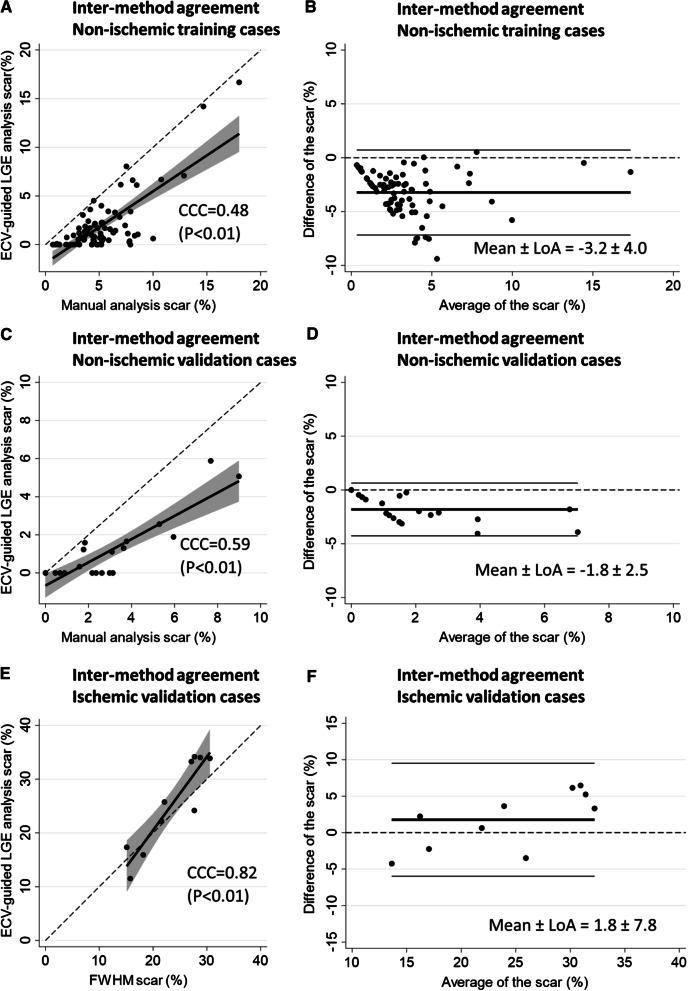
Scatter plot graphs and Bland–Altman plots of inter-method agreement of global scar amount (%) between the ECV-guided LGE analysis and the conventional methods in non-ischemic training cases, non-ischemic validation cases, and ischemic validation cases. **A** Scatter plot graph and **B** Bland–Altman plot of the inter-method agreement between the ECV-guided LGE analysis and the manual analysis in 80 non-ischemic cases. **C** Scatter plot graph and **D** Bland–Altman plot of the inter-method agreement between the ECV-guided LGE analysis and the manual analysis in 20 non-ischemic validation cases. **E** Scatter plot graph and **F** Bland–Altman plot of the inter-method agreement between the ECV-guided LGE analysis and the FWHM with manual correction in 10 ischemic validation cases. A moderate inter-method agreement was observed in non-ischemic cases. In ischemic cases, the agreement was excellent. *ECV* extracellular volume, *LGE* late gadolinium enhancement, *CCC* concordance correlation coefficient, *LoA* limits of agreement, *FWHM* full-width half-maximum

LGE scar was detected in all the cases on manual analysis while in 76.3% in ECV-guided LGE analysis (*P* < 0.01). The quantitative global scar amount (%) was significantly larger in manual analysis than that of the ECV-guided LGE analysis [4.5 (3.2–6.4) vs. 0.92 (0.1–2.1), *p* < 0.01]. The inter-method agreement of the global scar (%) between these two methods was fair (CCC = 0.48, *P* < 0.01) with the mean ± 95% LoA by the Bland–Altman plot of − 3.2 ± 3.9 (%) (Table 2; Fig. 5).

**Table 2:** Inter-method agreement of global scar amount (%) between the ECV-guided LGE analysis and the conventional methods in non-ischemic and ischemic cohorts

Disease and cohort name	Analysis method	N	LGE detection rate, n (%) (*P* value)	Scar amount (%), mean ± SD	Scar amount (%), median (IQR) (*P* value)	B-A plot mean ± LoA	CCC (*P* value)
Non-ischemic training cohort	ECV-guided LGE analysis versus Manual analysis	80	61 (76.3) versus 80 (100) (*p* < 0.01)	1.8 ± 2.9 versus 5.1 ± 3.0	0.92 (0.1–2.1) versus 4.5 (3.2–6.4) (*P* < 0.01)	− 3.2 ± 4.0	0.48 (*P* < 0.01)
Non-ischemic validation cohort	ECV-guided LGE analysis versus Manual analysis	20	10 (50.0) versus 18 (90.0) (*p* < 0.01)	1.1 ± 1.7 versus 2.9 ± 2.4	0.2 (0–1.6) versus 2.5 (1.2–3.7) (*P* < 0.01)	− 1.8 ± 2.5	0.59 (*P* < 0.01)
Ischemic validation cohort	ECV-guided LGE analysis versus FWHM	10	10 (100) versus 10 (100)	25.2 ± 8.5 versus 23.5 ± 5.7	25.0 (17.3–33.9) versus 24.6 (18.2–27.7) (*P* = 0.23)	1.8 ± 7.8	0.82 (*P* < 0.01)

Inter-method agreement was investigated in 80 cases of non-ischemic training cohort, 20 cases of non-ischemic validation cohort, and in 10 cases of ischemic validation cohort. A moderate correlation of scar amount (%) was observed between the ECV-guided LGE analysis and the manual analysis in the non-ischemic training cohort as well as in the validation cohort. In ischemic cases, the correlation was excellent

*ECV* extracellular volume, *LGE* late gadolinium enhancement, *SD* standard deviation, *IQR* interquartile range, *LoA* limits of agreement, *CCC* concordance correlation coefficient, *FWHM* full-width half-maximum

#### Reproducibility analysis in the non-ischemic training cohort

Forty cases were randomly selected for reproducibility analysis. Both inter- and intra-observer reproducibility presented better results in the ECV-guided LGE analysis than the manual analysis at the global level and at the segmental level. The intra-observer reproducibility of global scar (%) by ECV-guided LGE analysis was excellent (CCC = 0.94, *P* < 0.01), and was better than that by the manual scar analysis (CCC = 0.78, *P* < 0.01). The inter-observer reproducibility of global scar (%) by ECV-guided LGE analysis was excellent (CCC = 0.86, *P* < 0.01), and better than that by manual scar analysis (CCC = 0.73, *P* < 0.01). Bland–Altman analysis revealed tighter limits of agreement and smaller bias in ECV-guided LGE analysis, for both inter- and intra-observer assessments (Table 3 and Supplement 3 within the Additional file 1). In per-segmental scar (%) analysis, a similar trend of improved inter- and intra-observer reproducibility was observed for the ECV-guided LGE analysis as compared to the manual analysis (Supplement 4 within the Additional file 1).

**Table 3:** Intra- and inter-observer reproducibility of global scar amount (%) between the ECV-guided LGE analysis and the conventional methods in non-ischemic and ischemic cases

Disease	Reproducibility assessment	Analysis method	N	Scar amount (%), mean ± SD	Scar amount (%), median (IQR) (*P* value)	B-A plot mean ± LoA	CCC (*P* value)
Non-ischemic	Intra-observer	ECV-guided LGE analysis	40	2.9 ± 3.6 versus 2.7 ± 3.7	1.6 (0.8–3.5) versus 1.6 (0.4–3.7) (*P* = 0.34)	0.1 ± 2.5	0.94 (*P* < 0.01)
Non-ischemic	Intra-observer	Manual analysis	40	6.0 ± 3.4 versus 5.4 ± 3.4	5.2 (3.6–7.7) versus 4.6 (3.1–6.7) (*P* = 0.09)	0.6 ± 4.3	0.78 (*P* < 0.01)
Non-ischemic	Inter-observer	ECV-guided LGE analysis	40	2.9 ± 3.6 versus 2.3 ± 3.5	1.6 (0.8–3.5) versus 1.3 (0.2–3.3) (*P* = 0.05)	0.5 ± 3.6	0.86 (*P* < 0.01)
Non-ischemic	Inter-observer	Manual analysis	40	6.0 ± 3.4 versus 6.6 ± 3.4	5.2 (3.6–7.7) versus 6.3 (4.5–7.6) (*P* = 0.09)	− 0.6 ± 4.7	0.73 (*P* < 0.01)
Ischemic	Intra-observer	ECV-guided LGE analysis	10	25.2 ± 8.5 versus 26.6 ± 8.8	25.0 (17.3–33.9) versus 26.9 (17.5–35.2) (*P* = 0.01)	− 1.4 ± 3.6	0.96 (*P* < 0.01)
Ischemic	Intra-observer	FWHM	10	25.1 ± 5.9 versus 24.6 ± 5.6	25.0 (21.5–30.5) versus 23.9 (20.3–29.8) (*P* = 0.77)	0.6 ± 4.8	0.91 (*P* < 0.01)
Ischemic	Inter-observer	ECV-guided LGE analysis	10	25.2 ± 8.5 versus 28.3 ± 9.5	25.0 (17.3–33.9) versus 29.8 (21.4–37.1) (*P* < 0.01)	− 3.1 ± 4.3	0.91 (*P* < 0.01)
Ischemic	Inter-observer	FWHM	10	23.5 ± 5.7 versus 25.1 ± 5.9	24.6 (18.2–27.7) versus 25.0 (21.5–30.5) (*P* = 0.11)	− 1.7 ± 5.4	0.85 (*P* < 0.01)

Intra- and inter-observer reproducibility of global scar amount (%) was investigated in 40 cases of non-ischemic cases and in 10 cases of ischemic cases. In non-ischemic cases, both inter- and intra-observer reproducibility presented better results in the ECV-guided LGE analysis than the manual analysis. Bland–Altman analysis revealed tighter limits of agreement and smaller bias in ECV-guided LGE analysis, for both inter- and intra-observer assessments. In ischemic cases, all the intra- and inter-observer reproducibility of the global scar (%) were better in ECV-guided LGE analysis than the FWHM analysis, although the FWHM method was already presenting excellent intra-and inter-observer reproducibility. *ECV* extracellular volume, *LGE* late gadolinium enhancement, *SD* standard deviation, *IQR* interquartile range, *LoA* limits of agreement, *CCC* concordance correlation coefficient, *FWHM* full-width half-maximum

### ECV-guided LGE analysis validation in the non-ischemic validation cohort

The non-ischemic validation cohort presented similar trends of results with the non-ischemic training cohort. The ECV value corresponding to the high SI ROI and the global scar (%) were comparable to those of the non-ischemic training cohort [ECV value of the high SI ROI: 32.6 (27.8–35.4) vs. 34.0 ± 6.6 (%), *P* = 0.36, the global scar (%): 0.2 (0–1.6) vs. 0.92 (0.1–2.1) (%), *P* = 0.14]. The ECV cutoff of 31.5% achieved excellent scar/non-scar differentiation at the high SI ROI in the validation cohort (sensitivity 100%, specificity 81.8%, PPV 81.8%, and NPV 100%) while the nT1 cutoff of 1317 ms presented a fair differentiation in the validation cohort (sensitivity 33.3%, specificity 90.9%, PPV 75%, and NPV 62.5%) (Fig. 4C, D).The optimal threshold (n-SD) varied from 3 to 9SD but was lower than that of the non-ischemic training cohort [6 (4–7) vs. 7.3 ± 2.9 (SD), *P* = 0.02]. This n-SD threshold was associated with the scar amount (β = −0.53, *p* = 0.02). LGE scar detection rate was higher on manual analysis than on ECV-guided LGE analysis (90% vs. 50%, *P* < 0.01). The global scar amount (%) was significantly larger on manual analysis than on the ECV-guided LGE analysis [2.5 (1.2–3.7) vs. 0.2 (0–1.6), *P* < 0.01]. The inter-method agreement of the global scar (%) was fair (CCC = 0.59, *P* < 0.01) with the mean ± 95% LoA by the Bland–Altman plot of − 1.8 ± 2.5 (%). All of these trends were similar to those observed in the training cohort (Table 2; Fig. 5).

### ECV-guided LGE analysis validation in the ischemic cohort

The ECV value corresponding to the high SI ROI was 52.2 (49.1–54.3) (%) in the ischemic cohort. The optimal threshold (n-SD) was 3.5 (3–5) (SD). The inter-method agreement of the global scar (%) by the ECV-guided LGE analysis and the FWHM with manual correction was excellent [25.0 (17.3–33.9) vs. 24.6 (18.2–27.7), *P* = 0.23, CCC = 0.82, *P* < 0.01, the mean ± 95% LoA = 1.8 ± 7.8 (%)] (Table 2; Fig. 5). All the intra- and inter-observer reproducibility of the global scar (%) (Table 3 and Supplement 3 within the Additional file 1) were better or comparable in ECV-guided LGE analysis than the conventional analysis. In the per-segmental scar (%) analysis, similar trends were observed in the inter-method agreement and the reproducibility (Supplement 4 within the Additional file 1).

## Discussion

In this study, we have presented an ECV-guided LGE analysis method that uses the ECV map as a guide to determine the optimal LGE n-SD threshold in individual cases. This method was developed in a cohort of women with or at-risk of HIV which presented an LGE pattern of diffuse and patchy non-ischemic cardiomyopathy. The ECV cutoff of 31.5% successfully differentiated scar from non-scar, achieving high sensitivity, specificity, NPV, and PPV. LGE n-SD threshold was optimized using the ECV map as a reference, which also contributed to the high reproducibility of this method. The selected n-SD threshold ranged from 3 to 18SD. The current study also suggested that the manual LGE assessment on non-ischemic diffuse and patchy fibrosis may be overestimating the scar amount. Overall, the ECV-guided LGE analysis was a more robust LGE quantification than the conventional quantification method for non-ischemic LGE cases. The robustness of ECV-guided LGE analysis was also confirmed in two validation cohorts of non-ischemic and ischemic cases.

The development of ECV criteria involved three steps of considerations. First, we selected ECV, not nT1, to guide the LGE analysis. This was based on the better performance of ECV to differentiate scar/non-scar than nT1 (Fig. 4). In addition, the correlation between ECV and SI was stronger than that of between nT1 and SI (ECV and SI: *r*_s_ = 0.35, *P* < 0.01; nT1 and SI: *r*_s_ = 0.27, *P* = 0.02), which was in line with the literature that reported a significant linear correlation between ECV and histological collagen volume fraction (CVF), but no significant correlation between nT1 and CVF [32, 43–46]. The greater robustness of ECV relative to nT1 values at different imaging parameters and scanner field strengths [33, 34] further supported our decision. Second, there was a discussion on the ECV cutoff values which corresponded to the LGE. Such ECV cutoff values specific for the HIV patients have not been investigated so far and therefore, the ECV criteria was developed from our 80 training cases. Indeed, our ECV cutoff of 31.5% was consistent with other publications. In a cohort with myocardial infarction or hypertrophic cardiomyopathy (HCM) patients, the ECV cutoff value for LGE was 32% [47]. In a diastolic cardiomyopathy cohort, CVF cutoff of 12% (which calculates to the ECV value of 30.5%) corresponded to the LGE [30] while in HCM, the CVF cutoff was 15% [48]. ECV values of remote myocardium and LGE scar area were also referenced from HCM (28 ± 4% vs. 30 ± 5%, *P* < 0.001) [49], non-ischemic cardiomyopathy (26 ± 3% vs 37 ± 6%, *P* < 0.001) [50], and myocardial infarction (27 ± 3% vs 51 ± 8%, *P* < 0.001) [50]. Thirdly, there was a potential trade-off of false-positive or false-negative with regard to the single cutoff strategy. Such misclassification was observed in 9 cases (11.3%), typically when ECV values were close to the ECV cutoff value of 31.5% (averaged ECV was 31.6 ± 1.9%). A lower ECV cutoff to achieve a higher sensitivity or a higher ECV cutoff to achieve a higher specificity may be considered, although in such situations, the counterpart of specificity or sensitivity will be compromised. Indeed, a lower ECV cutoff of ECV = 30% achieved an excellent NPV with sensitivity, specificity, PPV, and NPV of 98.0%, 63.3%, 81.7%, and 95.0%, respectively, while a higher ECV cutoff of ECV = 35% achieved an excellent PPV with sensitivity, specificity, PPV, and NPV of 58.0%, 100%, 100%, and 58.8%, respectively. In spite of the excellent performance of ECV criteria as shown in our manuscript, when the observer’s visual decision is clearly against the ECV value, it may be important to re-evaluate the quality of the ECV map and prioritize the observer’s decision.

Our study presented multiple advantages of ECV-guided LGE analysis for the non-ischemic LGE quantification. First, the personalized optimization of the LGE n-SD cut-off enables its application to different pathophysiologies and to assess disease progression. Considering the broad range of n-SD threshold applied, a fixed semi-quantitative threshold was not the optimal choice for our cohort. This finding is in line with the publication that the individual optimization of the LGE cut-off was more effective than a fixed cut-off of 2-SD or 6-SD in a cohort of HCM [51]. Second, given the ECV map as a guide, the observers could rationally determine the absence of a scar (Fig. 3, Case 2). In our study, this contributed to the difference in scar detection rate or scar amount between the proposed method and the manual analysis. Utilization of the ECV map as a guide especially helps the analysts when quantifying the LGE scar with a relatively unknown distribution [6, 7, 12–16]. Third, the excellent reproducibility of ECV-guided LGE analysis is an advantage in the systematic detection of small changes in scar size for the monitoring and management of non-ischemic patients, as well as to determine the prognostic risk of patients more accurately. Many non-ischemic disease groups present relatively small LGE scar amounts as compared to ischemic cardiomyopathy and HCM [1–4]. The clinical impact of its per-unit change of LGE may likely be different among etiologies. In this regard, ECV-guided LGE analysis is sensitive to a small change in LGE scar size so that the corresponding change in myocardial disease may be detected more sensitively. Additionally, reproducibility is a key determinant of required sample sizes for clinical trials. ECV-guided LGE analysis potentially allows a substantial reduction in the sample size, which is a great benefit for a clinical study [20, 52, 53]. The higher reproducibility of semi-quantitative LGE scar analysis than manual has not been explored in patients with HIV except our study, but a similar trend has been reported in studies with different non-ischemic etiologies such as myocarditis [5] and HCM [9, 20]. We may reconsider the significance of the semi-quantitative LGE assessment in non-ischemic cases. In our study, the results suggested an overestimation of scar in manual analysis. This may be different across different pathologies and for different readers, but it brings to light the difficulty in reproducibly obtaining scar amounts in non-ischemic cardiomyopathy.

The validation studies on the non-ischemic and ischemic cohorts proved the robustness of the ECV-guided LGE analysis. The non-ischemic validation cohort presented similar trends as the training cohort in the inter-method analysis of the scar (%). This again suggested that the manual LGE assessment on non-ischemic cases may be overestimating the scar amount. In the ischemic cohort, ECV-guided LGE analysis achieved high reproducibility. Although in this cohort, the conventional method of FWHM with manual correction was already presenting excellent performance and therefore, there was only a small room for the proposed method to improve reproducibility. This was derived from the high SI and the well-known distribution of the ischemic scar. The ECV-guided LGE analysis was feasible in the ischemic cases but did not necessarily surpass the conventional method.

Several study limitations deserve discussion. First, ECV-guided LGE analysis cannot be performed when either the LGE or T1 map image is missing or in cases of poor image quality, which occurred in 19 cases (19%) and were excluded from this study. In such cases, manual scar analysis was performed. Second, the n-SD threshold selection was performed with only one slice in the mid-LV due to the current technical limitation of T1 mapping. 3D T1 mapping methods [54, 55] with a matched slice position may give a more defined n-SD threshold selection at each slice level, which may then be applied to higher resolution LGE images. Third, the performance of ECV criteria was not compared with pathology, since myocardial biopsy was not available in the study protocol as well as obtaining the myocardium from the same location as suggested in the MRI images was not practical. Fourth, the ECV-guided LGE analysis requires gadolinium-based contrast administration, which is not applicable to the patients with contraindication to the agent. Native T1 map analysis may be utilized in such cases to synthesize corresponding LGE area, admitting the moderate scar differentiation performance of nT1 (Fig. 4). Finally, our ECV-guided LGE method was developed in a unique cohort of women with or at risk for HIV infection and validated in a non-ischemic and an ischemic cohort but with a small number of cases. Further validation in a larger number of participants with multiple etiologies may help validate the method further.

In conclusion, ECV-guided LGE analysis is a robust and comprehensive method of scar burden and distribution assessment in participants with diffuse and patchy fibrosis, achieving both higher intra- and inter-observer reproducibility as compared to manual analysis.

## Supplementary Information

**Additional file 1.** (**Supplement 1**) Summary of the ischemic validation cohort and the MRI imaging parameters. (**Supplement 2**) MRI imaging parameters for the non-ischemic cohort. (**Supplement 3**)Intra- and inter-observer reproducibility of global scar amount (%) between the ECV-guided LGE analysis and the conventional methods in non-ischemic and ischemic cases. (**Supplement 4**) Results of segmental scar amount (%) in non-ischemic and ischemic cases.

## Data Availability

The datasets used and/or analyzed during the current study are available from the corresponding author on reasonable request.

## Abbreviations

LGE: Late gadolinium enhancement
ECV: Extracellular volume
HIV: Human immunodeficiency virus
ROI: Region of interest
SI: Signal intensity
LV: Left ventricle
SD: Standard deviation
CCC: Concordance correlation coefficient
PSIR: Phase-sensitive inversion recovery
MOLLI: Modified Look-Locker Imaging
ROC: Receiver-operating curve
AUC: Area under the curve
PPV: Positive-predictive value
NPV: Negative-predictive value
LoA: Limits of agreement
FWHM: Full-width half-maximum
CVF: Collagen volume fraction
HCM: Hypertrophic cardiomyopathy
