# Supplementary material for: Extracellular volume-guided late gadolinium enhancement analysis for non-ischemic cardiomyopathy: The Women’s Interagency HIV Study
Source: BMC Med Imaging. 2021 Jul 27;21:116. doi: 10.1186/s12880-021-00649-6 (PMC8314536; doi:10.1186/s12880-021-00649-6)
Supplement: Supplementary file 1 — Additional file 1. (Supplement 1) Summary of the ischemic validation cohort and the MRI imaging parameters. (Supplement 2) MRI imaging parameters for the non-ischemic cohort. (Supplement 3)Intra- and inter-observer reproducibility of global scar amount (%) between the ECV-guided LGE analysis and the conventional methods in non-ischemic and ischemic cases. (Supplement 4) Results of segmental scar amount (%) in non-ischemic and ischemic cases. [file 12880_2021_649_MOESM1_ESM.docx]

**Supplemental Material**

(Supplement 1) Summary of the ischemic validation cohort and the MRI imaging parameters

Ten cases of ischemic heart failure were selected from a separate cohort study titled “Combination of Mesenchymal and c-kit+ Cardiac Stem Cells As Regenerative Therapy for Heart Failure (CONCERT-HF)”, which involved cardiac MRI protocol of LGE and T1 mapping (1). In this study, the T1 mapping image acquisition was optional, and accordingly, a limited number of cases were available for both LGE and T1 mapping images. The participant’s characteristics were summarized in Table 1. The MRI images were acquired on 1.5T scanners (Achieva; Philips Healthcare, Best, the Netherlands, or Signa HDxt; General Electric, Milwaukee, WI) (1). The representative MRI imaging parameters for LGE and T1 mapping were as follows.

LGE image acquisition: A standard segmented ‘fast low-angle shot’ two-dimensional inversion-recovery gradient echo sequence LGE PSIR sequence  was used with the following typical imaging parameters: flip angle=20°; TR/TE=7.6/3.8ms; pixel bandwidth 383Hz/pixel; matrix = 180 × 155; voxel size = 1.4 × 1.4 × 8 mm; 2mm gap. Approximately 10 slices were acquired, each within 15 seconds breath hold. The inversion time (TI) was adjusted in each scan to null the signal of the remote myocardium.

T1 mapping image acquisition: Modified Look-Locker Imaging (MOLLI) sequence was used with the following typical imaging acquisition parameters: flip angle = 35°; TR/TE = 3.7/0 ms; pixel bandwidth 781 Hz/pixel; matrix = 256 × 193; voxel size = 1.1 ×1.1 × 8 mm. Automatic in-line motion correction was performed to align images acquired at different inversion times on the MR console (2). To further reduce any remaining misregistration between these images, manual motion correction was performed offline before pixel-wise T1 maps were generated using a non-linear least-square curve fitting (3).

**References**

1. Bolli R, Hare JM, March KL, Pepine CJ, Willerson JT, Perin EC, Yang PC, Henry TD, Traverse JH, Mitrani RD, et al. Rationale and design of the CONCERT-HF trial (combination of mesenchymal and c-kit + cardiac stem cells as regenerative therapy for heart failure). Circ Res (2018) 122:1703–1715. doi:10.1161/CIRCRESAHA.118.312978

2. Xue H, Shah S, Greiser A, Guetter C, Littmann A, Jolly M-P, Arai AE, Zuehlsdorff S, Guehring J, Kellman P. Motion correction for myocardial T1 mapping using image registration with synthetic image estimation. Magn Reson Med (2012) 67:1644–55. doi:10.1002/mrm.23153

3. Messroghli DR, Greiser A, Fröhlich M, Dietz R, Schulz-Menger J. Optimization and validation of a fully-integrated pulse sequence for modified look-locker inversion-recovery (MOLLI) T1 mapping of the heart. J Magn Reson Imaging (2007) 26:1081–6. doi:10.1002/jmri.21119

(Supplement 2) MRI imaging parameters for the non-ischemic cohort

LGE image acquisition: A standard segmented ‘fast low-angle shot’ two-dimensional inversion-recovery gradient echo sequence LGE PSIR sequence  was used with the following typical imaging parameters: flip angle=25°; TR/TE=6.1/3.0ms; pixel bandwidth 229Hz/pixel; matrix = 200 × 133; voxel size = 1.97 × 2.0 × 10 mm; ; 1mm gap. Approximately 10 slices were acquired, each within 15 seconds breath hold. The inversion time (TI) was adjusted in each scan to null the signal of the remote myocardium.

T1 mapping image acquisition: Modified Look-Locker Imaging (MOLLI) sequence provided by the MR scanner vendor or the recently proposed 5s(3s)3s and 4s(1s)3s(1s)2s schemes (2) was used with the following typical imaging acquisition parameters: flip angle = 20°; TR/TE = 1.84/0.68 ms; pixel bandwidth 1082 Hz/pixel; matrix = 152 × 150; voxel size = 1.97 × 2.0 × 10 mm. Automatic in-line motion correction was performed to align images acquired at different inversion times on the MR console [1]. To further reduce any remaining mis-registration between these images, manual motion correction was performed offline before pixel-wise T1 maps were generated using a non-linear least-square curve fitting [2].

**References**

1. Xue H, Shah S, Greiser A, Guetter C, Littmann A, Jolly M-P, Arai AE, Zuehlsdorff S, Guehring J, Kellman P. Motion correction for myocardial T1 mapping using image registration with synthetic image estimation. Magn Reson Med (2012) 67:1644–55. doi:10.1002/mrm.23153

2. Messroghli DR, Greiser A, Fröhlich M, Dietz R, Schulz-Menger J. Optimization and validation of a fully-integrated pulse sequence for modified look-locker inversion-recovery (MOLLI) T1 mapping of the heart. J Magn Reson Imaging (2007) 26:1081–6. doi:10.1002/jmri.21119

(Supplement 3) Intra- and inter-observer reproducibility of global scar amount (%) between the ECV-guided LGE analysis and the conventional methods in non-ischemic and ischemic cases.


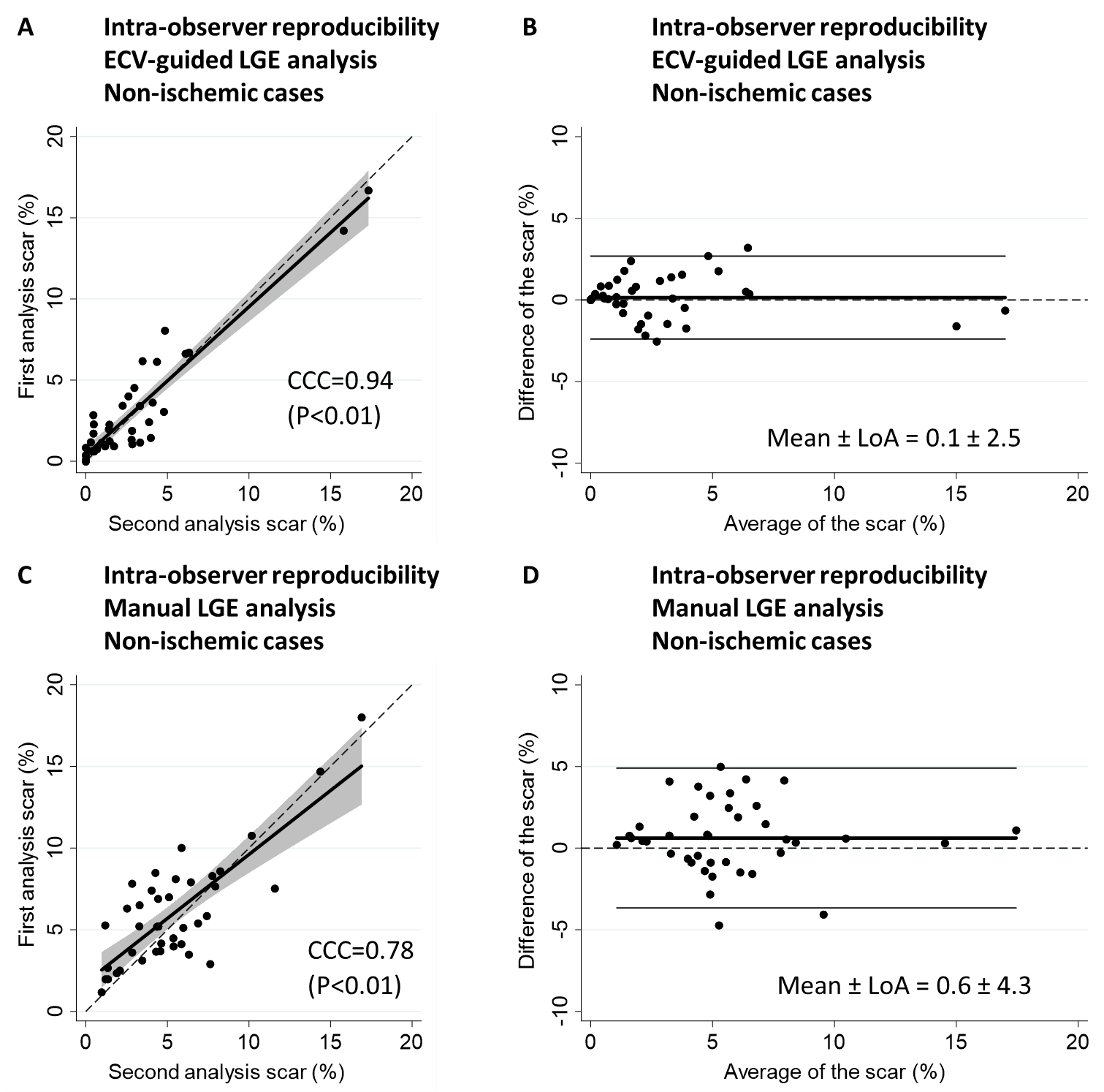


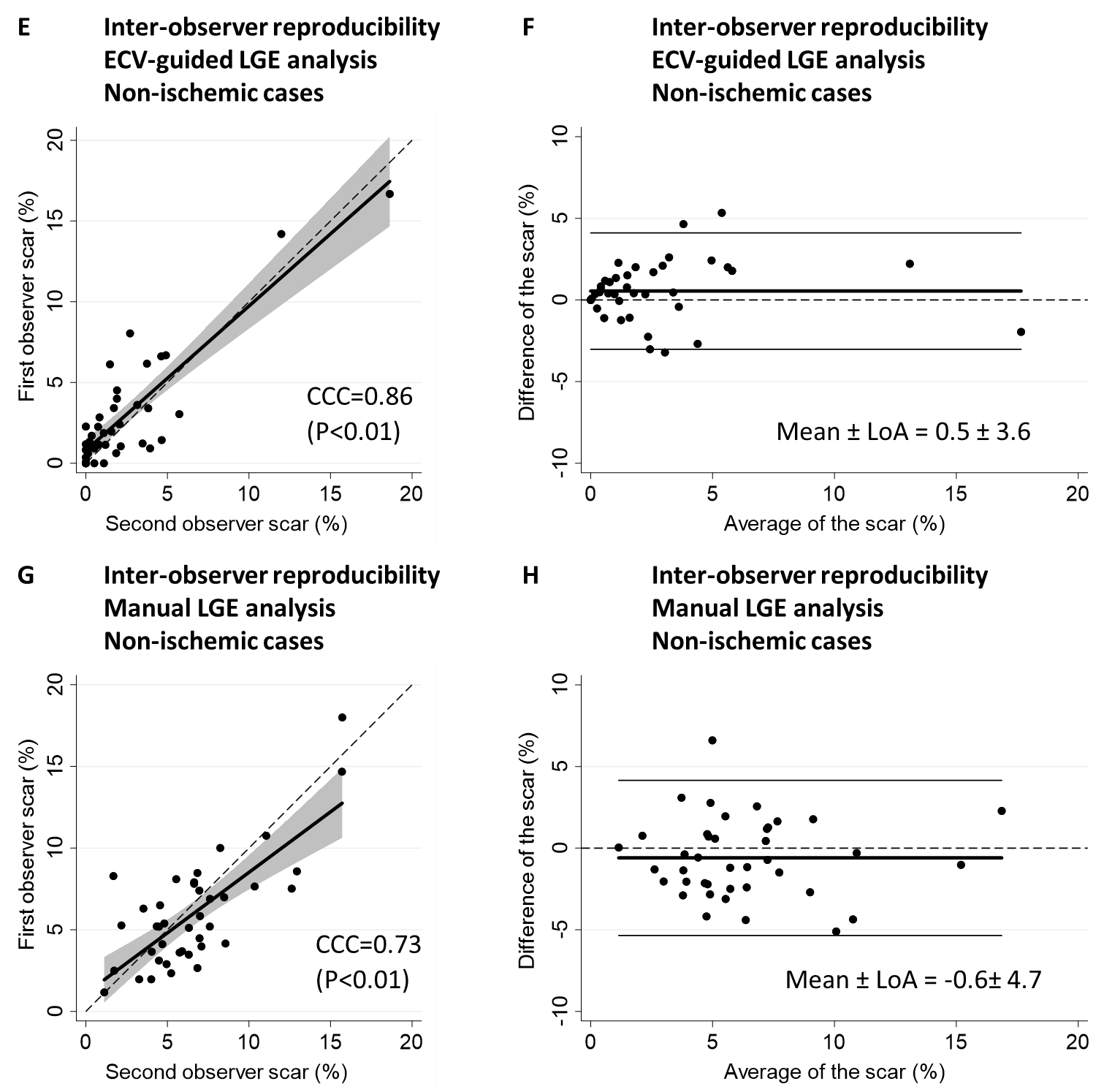


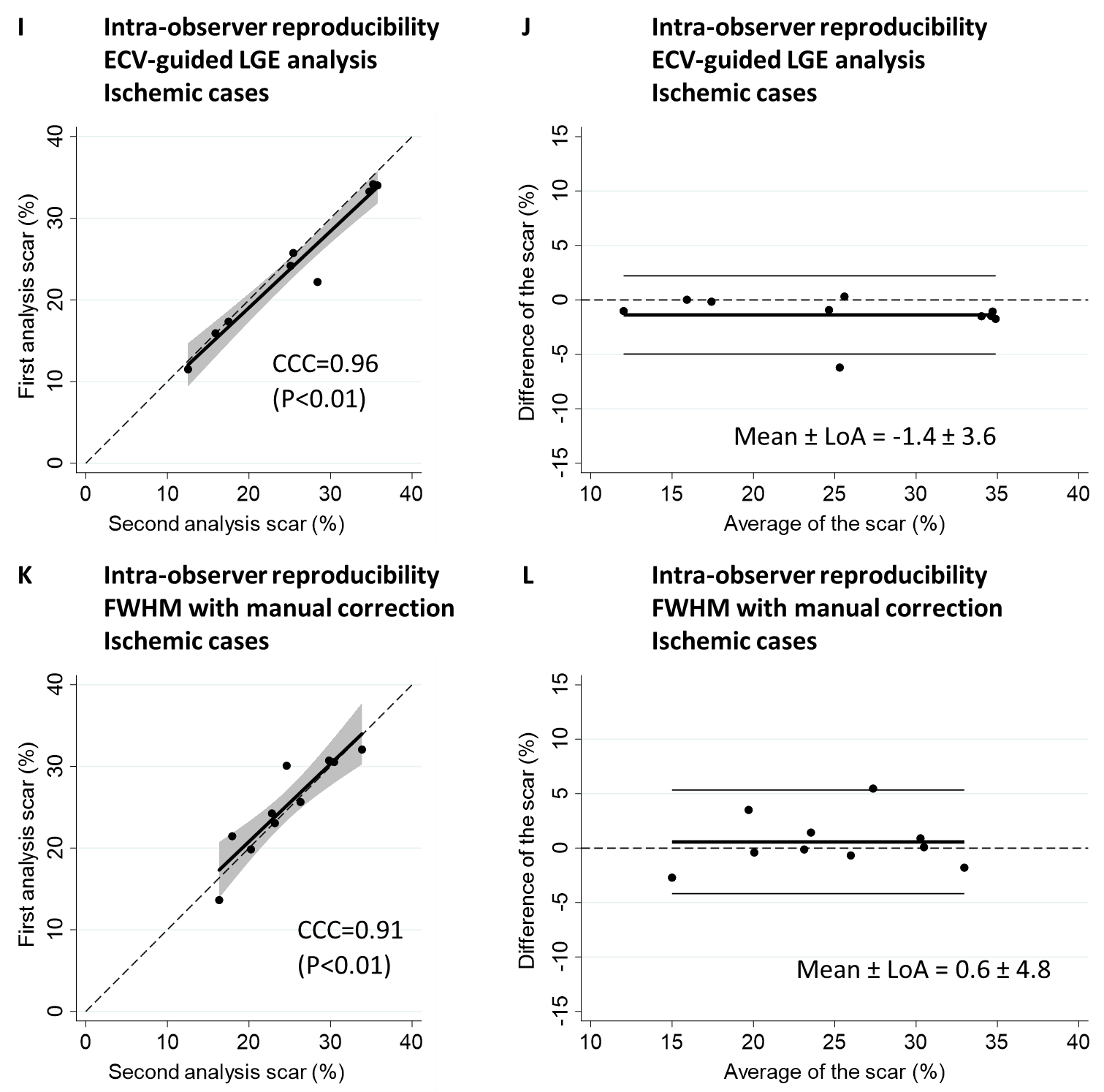


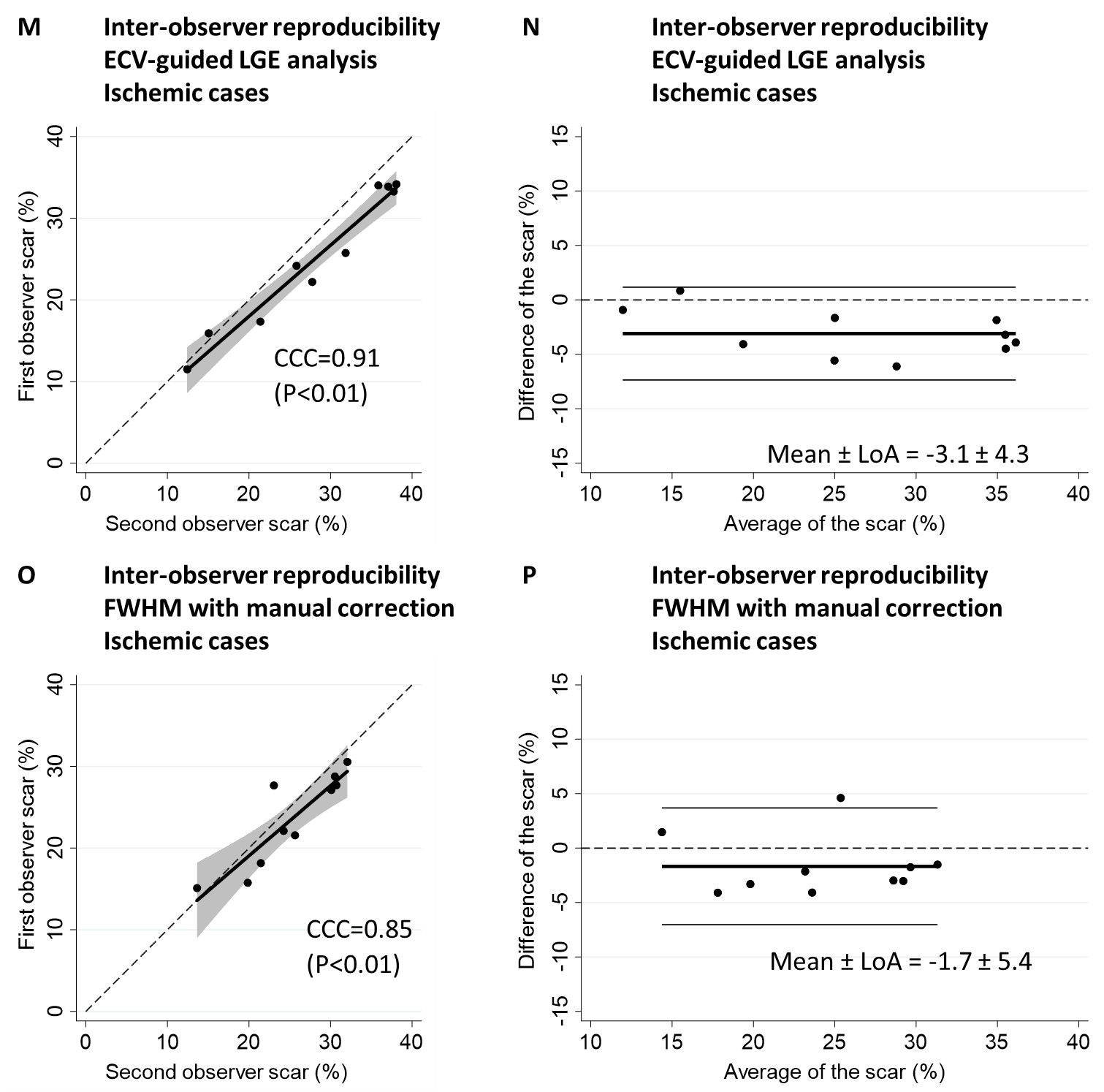


**A**, Scatter plot graph and **B**, Bland-Altman plot of the intra-observer reproducibility by ECV-guided LGE analysis in 40 non-ischemic cases. **C**, Scatter plot graph and **D**, Bland-Altman plot of the intra-observer reproducibility by conventional manual LGE analysis in 40 non-ischemic cases. **E**, Scatter plot graph and **F**, Bland-Altman plot of the inter-observer reproducibility by ECV-guided LGE analysis in 40 non-ischemic cases. **G**, Scatter plot graph and **H**, Bland-Altman plot of the inter-observer reproducibility by conventional manual LGE analysis in 40 non-ischemic cases. **I**, Scatter plot graph and **J**, Bland-Altman plot of the intra-observer reproducibility by ECV-guided LGE analysis in 10 ischemic cases. **K**, Scatter plot graph and **L**, Bland-Altman plot of the intra-observer reproducibility by conventional FWHM with manual correction LGE analysis in 10 ischemic cases. **M**, Scatter plot graph and **N**, Bland-Altman plot of the inter-observer reproducibility by ECV-guided LGE analysis in 10 ischemic cases. **O**, Scatter plot graph and **P**, Bland-Altman plot of the inter-observer reproducibility by conventional FWHM with manual correction LGE analysis in 10 ischemic cases. ECV = extracellular volume. LGE = late gadolinium enhancement. CCC = concordance correlation coefficient. LoA = limits of agreement. FWHM = full-width half-maximum.

(Supplement 4) Results of segmental scar amount (%) in non-ischemic and ischemic cases

Supplement 4-1. Inter-method agreement of segmental scar amount (%) between the ECV-guided LGE analysis and the conventional methods in non-ischemic training cohort, non-ischemic validation cohort, and ischemic validation cohort.

| Disease and cohort name | Analysis method | N | Scar amount (%), mean ± SD | Scar amount (%), median (IQR)  (*P* value) | B-A plot  mean ± LoA | CCC  (*P* value) |
| --- | --- | --- | --- | --- | --- | --- |
| Non-ischemic training cohort | ECV-guided LGE analysis vs. Manual analysis | 1280 | 1.3 ± 3.6 vs. 3.9 ± 4.6 | 0 (0 – 0.7) vs.  2.4 (0.2 – 6.0)  (*P*<0.01) | -2.6 ± 6.9 | 0.54 (*P*<0.01) |
| Non-ischemic validation cohort | ECV-guided LGE analysis vs. Manual analysis | 320 | 1.3 ± 3.8 vs. 2.1 ± 3.9 | 0 (0 – 0.6) vs.  0 (0 – 2.7)  (*P*<0.01) | -0.8 ± 7.4 | 0.51 (*P*<0.01) |
| Ischemic validation cohort | ECV-guided LGE analysis vs. FWHM with manual correction | 160 | 26.4 ± 21.9 vs. 24.4 ± 20.6 | 25.0 (3.7 – 45.4) vs. 23.1 (3.8 – 40)  (*P*<0.01) | 2.0 ± 13.6 | 0.94 (*P*<0.01) |

Inter-method agreement was investigated in 80 cases of non-ischemic training cohort, 20 cases of non-ischemic validation cohort, and in 10 cases of ischemic validation cohort. A moderate correlation of scar amount (%) was observed between the ECV-guided LGE analysis and the manal analysis in both non-ischemic cohorts. In ischemic cohort, the correlation was excellent. ECV = extracellular volume. LGE = late gadolinium enhancement. SD = standard deviation. IQR = interquartile range. LoA = limits of agreement. CCC = concordance correlation coefficient. FWHM = full-width half maximum.

Supplement 4-2. Intra- and inter- observer reproducibility of segmental scar amount (%) between the ECV-guided LGE analysis and the conventional methods in non-ischemic and ischemic cases.

| Disease | Reproducibility assessment | Analysis method | N | Scar amount (%), mean ± SD | Scar amount (%), median (IQR)  (*P* value) | B-A plot mean ± LoA | CCC (*P* value) |
| --- | --- | --- | --- | --- | --- | --- | --- |
| Non-ischemic | Intra-observer | ECV-guided LGE analysis | 640 | 2.0 ± 4.4 vs. 2.2 ± 4.7 | 0.2 (0 – 1.8) vs. 0.3 (0 – 2.0)  (*P*=0.04) | -0.2 ± 4.1 | 0.89 (*P*<0.01) |
| Non-ischemic | Intra-observer | Manual analysis | 640 | 4.6 ± 5.0 vs.  4.4 ± 6.1 | 2.9 (0.6 – 7.3) vs. 1.7 (0 – 6.9)  (*P*<0.01) | 0.2 ± 9.0 | 0.68 (*P*<0.01) |
| Non-ischemic | Inter-observer | ECV-guided LGE analysis | 640 | 2.0 ± 4.4 vs.  2.6 ± 6.1 | 0.2 (0 – 1.8) vs. 0.2 (0 – 2.0)  (*P*=0.06) | -0.6 ± 7.6 | 0.69 (*P*<0.01) |
| Non-ischemic | Inter-observer | Manual analysis | 640 | 4.6 ± 5.0 vs. 5.7 ± 6.0 | 2.9 (0.6 – 7.3) vs. 4.0 (1.2 – 8.2)  (*P*<0.01) | -1.0 ± 11.0 | 0.48 (*P*<0.01) |
| Ischemic | Intra-observer | ECV-guided LGE analysis | 160 | 26.4 ± 21.9 vs. 28.3 ± 23.6 | 25.0 (3.7 – 45.4) vs. 25.7 (4.1 – 47.3) (*P*<0.01) | -2.0 ± 8.9 | 0.98 (*P*<0.01) |
| Ischemic | Intra-observer | FWHM | 160 | 27.2 ± 22.4 vs. 26.7 ± 22.2 | 22.7 (7.6 – 42.1) vs. 22.9 (6.7 – 41.2) (*P*=0.68) | 0.56 ± 19.9 | 0.90 (*P*<0.01) |
| Ischemic | Inter-observer | ECV-guided LGE analysis | 160 | 26.4 ± 21.9 vs. 30.2 ± 25.7 | 25.0 (3.7 – 45.4) vs. 25.8 (5.7 – 47.9) (*P*<0.01) | -3.8 ± 20.4 | 0.89 (*P*<0.01) |
| Ischemic | Inter-observer | FWHM | 160 | 24.4 ± 20.6 vs. 27.2 ± 22.4 | 23.1 (3.8 – 40) vs. 22.7 (7.6 – 42.1) (*P*<0.01) | -2.8 ± 23.4 | 0.84 (*P*<0.01) |

Intra- and inter- observer reproducibility of global scar amount (%) was investigated in 640 segments from 40 cases of non-ischemic cohort and in 160 segments from 10 cases of ischemic cohort. Both inter- and intra-observer reproducibility presented better results in the ECV-guided LGE analysis than the manual analysis. Bland-Altman analysis revealed tighter limits of agreement and smaller bias in ECV-guided LGE analysis, for both inter- and intra-observer assessments. In ischemic cases, all the intra- and inter- observer reproducibility of the global scar (%) were better in ECV-guided LGE analysis than the conventional analysis, although the conventional method was already presenting excellent intra-and inter- observer reproducibility. ECV = extracellular volume. LGE = late gadolinium enhancement. SD = standard deviation. IQR = interquartile range. LoA = limits of agreement. CCC = concordance correlation coefficient. FWHM = full-width half maximum.
